# Using Facebook Advertising to Recruit Representative Samples: Feasibility Assessment of a Cross-Sectional Survey

**DOI:** 10.2196/14021

**Published:** 2019-08-19

**Authors:** Lance Garrett Shaver, Ahmed Khawer, Yanqing Yi, Kris Aubrey-Bassler, Holly Etchegary, Barbara Roebothan, Shabnam Asghari, Peizhong Peter Wang

**Affiliations:** 1 Division of Community Health and Humanities Faculty of Medicine Memorial University of Newfoundland St John's, NL Canada; 2 Primary Healthcare Research Unit Discipline of Family Medicine Memorial University of Newfoundland St John's, NL Canada; 3 Clinical Epidemiology Faculty of Medicine Memorial University of Newfoundland St John's, BC Canada; 4 Dalla Lana School of Public Health University of Toronto Toronto, ON Canada; 5 School of Public Health and Management Weifang Medical University Weifang China

**Keywords:** Facebook, health surveys, Canada, research subject recruitment, social media, internet, online recruitment

## Abstract

**Background:**

Facebook has shown promise as an economical means of recruiting participants for health research. However, few studies have evaluated this recruitment method in Canada, fewer still targeting older adults, and, to our knowledge, none specifically in Newfoundland and Labrador (NL).

**Objective:**

This study aimed to assess Facebook advertising as an economical means of recruiting a representative sample of adults aged 35 to 74 years in NL for a cross-sectional health survey.

**Methods:**

Facebook advertising was used to recruit for a Web-based survey on cancer awareness and prevention during April and May 2018; during recruitment, additional advertisements were targeted to increase representation of demographics that we identified as being underrepresented in our sample. Sociodemographic and health characteristics of the study sample were compared with distributions of the underlying population to determine representativeness. Cramer V indicates the magnitude of the difference between the sample and population distributions, interpreted as small (Cramer V=0.10), medium (0.30), and large (0.50). Sample characteristics were considered representative if there was no statistically significant difference in distributions (chi-square *P*>.01) or if the difference was small (V≤0.10), and practically representative if 0.10<V≤0.20. The cost per recruit of Facebook advertising was compared with a quote for a random digit dialing (RDD)–recruited postal survey to determine if this method was economical.

**Results:**

Facebook advertising is feasible and economical to conduct survey research, reaching 34,012 people, of which 2067 clicked on the ad, for a final sample size of 1048 people at Can $2.18 per recruit versus the quoted Can $23,316.05 for 400 recruits (Can $35.52 per recruit) via RDD. The sample was representative of rural and urban geography (*P*=.02; V=0.073), practically representative of age (*P*=.003; V=0.145) and income (*P*<.001; V=0.188), and over-representative of women (*P*<.001; V=0.507) and higher levels of education (*P*<.001; V=0.488). The sample was representative of the proportion of people with a regular health care provider (*P*=.94; V=0.025), diabetes prevalence (*P*=.002; V=0.096), and having had a colonoscopy or sigmoidoscopy (*P*=.27; V=0.034), and it was practically representative of smoking status (*P*<.001; V=0.14), and body mass index (*P*<.001; V=0.135). The sample was not representative of arthritis prevalence (*P*<.001; V=0.573), perceived health (*P*<.001; V=0.384), or time since last seasonal flu shot (*P*<.001; V=0.449).

**Conclusions:**

Facebook advertising offers an easy, rapid, and economical means to recruit a partially representative (representative or practically representative of 8 of the 13 characteristics studied) sample of middle-aged and older adults for health survey research. As Facebook uses a nonrandom targeting algorithm, caution is warranted in its applications for certain types of research.

## Introduction

Population-based survey research relies on the recruitment of participants and aims for collected samples to be representative of the underlying target population. However, traditional survey research can be limited by high recruitment costs, low response rates, and considerable time and personnel demands [1]. With reductions in landline telephone use, even traditional methods, such as random digit dialing (RDD), are challenged in recruiting representative samples of the population [2]. Only 72% of the Canadian households have landlines [3,4], limiting the ability of telephone recruitment in achieving representative samples [3].

Facebook has increasingly gained attention as a tool to recruit participants for research [5]. We propose that many of the aforementioned limitations associated with traditional methods of recruitment can be resolved through Web-based recruitment, specifically Facebook advertising. Facebook currently stands as one of the most popular social media platforms, with over 2 billion users across the globe [6]. Among internet users aged 18 years and older in Canada, the Atlantic provinces (which include Newfoundland and Labrador, ie, NL) have the highest Facebook usage, with 94% using Facebook and 73% using it daily [7]. Even in the age group that uses Facebook the least (individuals aged 55 years and older), 78% of those who use the internet said they used Facebook, with 52% using it daily [8]. With a broad geographic spread and unique health challenges, NL offers a unique population to study Facebook recruitment. These features make traditional recruitment with either probabilistic or nonprobabilistic sampling methods challenging, particularly because of the large geographic spread. Facebook also offers additional benefits with its ability to target advertisements to preferentially reach people based on demographics, location, interests, and behaviors [9].

Limited research has examined the use of Facebook for survey recruitment in Canada and, to our knowledge, none in NL. Several studies, including systematic reviews, have suggested that Facebook is economical and can yield representative survey samples [1,5]. Several reviews have found only limited evidence for Facebook recruitment of older adults, and thus it remains unclear whether a representative sample of adults in this age group can be recruited by this method [5,10]. The objective of our study is to investigate whether Facebook advertising can be a feasible (timely, economical, and with minimal human resource commitment) means of recruiting a representative sample of adults aged 35 to 74 years for a health research survey in NL. Our study will further seek to provide clarity in the matter of whether older populations can be effectively reached and recruited through Facebook. This investigation is a supplement to our primary project, a cross-sectional study examining cancer awareness, beliefs, and prevention-related health behaviors in the NL population.

## Methods

### Study Design

To participate in this survey, participants must have (1) been aged 35 to 74 years, (2) been living in NL for 2 or more years, and (3) provided consent to participate in the survey.

To ensure transparency in the study design and recruitment process, we followed the reporting guidelines for the checklist for reporting results of internet surveys, reporting these details throughout our methods [11]. A Facebook page was created for the study as a medium for posting advertisements, responding to inquiries, and for knowledge translation once the research was completed. No incentives were offered for participation. The central theme of our survey was cancer awareness and prevention, focusing largely on health behaviors. Google Forms was used to host the open survey and allow for automatic capture of responses into a spreadsheet. We did not use item randomization or adaptive questioning. There were 134 items and 13 screens, 9 for the survey, 1 for the introduction and consent, 2 for eligibility, and 1 to thank participants and offer the opportunity to contact us or provide feedback. No completeness check was used, but participants could return to previous pages and change responses.

### Recruitment

**Figure 1:**
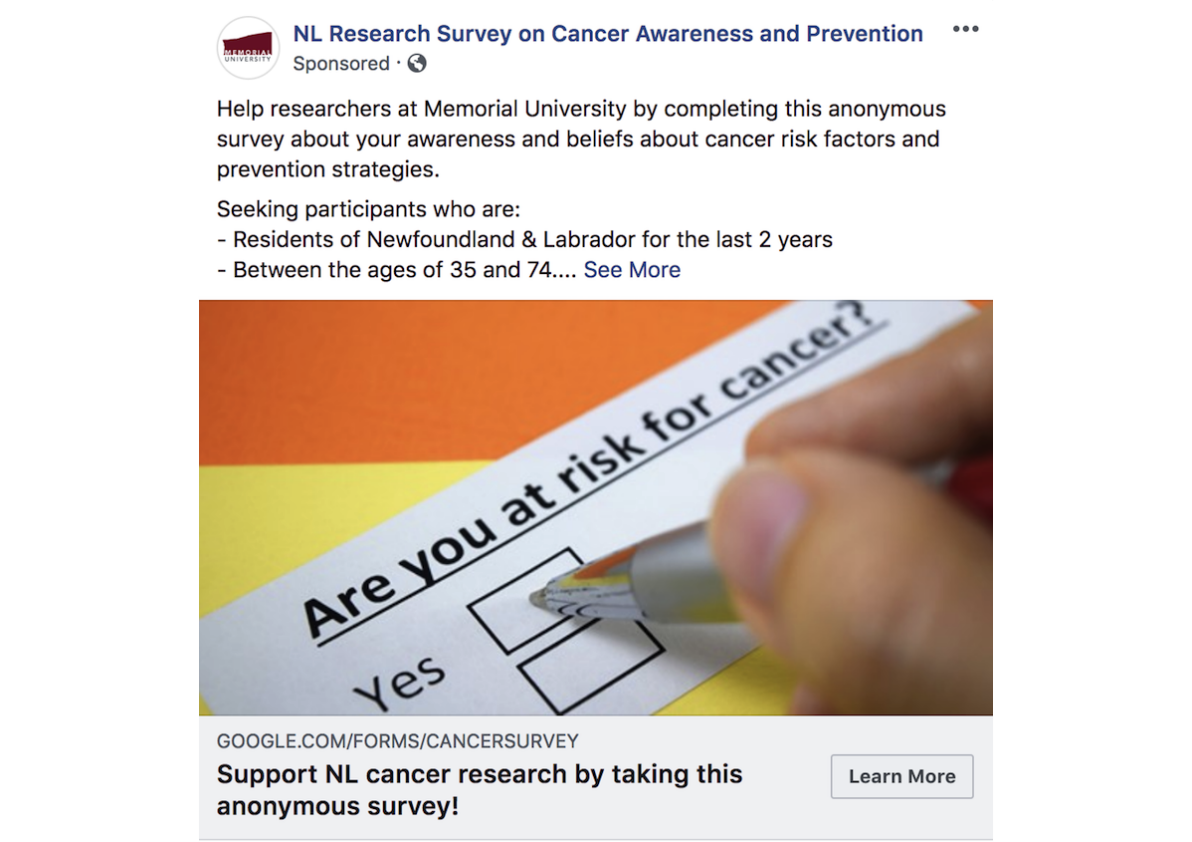
Generic Facebook advertisement.

The Facebook platform captures sociodemographic information that is self-reported by users (Facebook requires self-reporting for age and gender but not for education) and geographic location is captured based on data provided by the user, data from their device, and their Facebook activity [12]. Initially, we had just 1 generic advertisement (see Figure 1) targeted to all individuals aged 35 to 65 years and older (the maximum age that can be specified for Facebook ad targeting is *65 years and older*) living in NL. While recruitment was underway, we regularly evaluated the distribution of respondent demographics to assess if any were under-represented in our sample. We noticed that (1) men, (2) individuals from rural NL, (3) individuals aged 35 to 44 years, and (4) individuals with lower levels of education were under-represented in our sample. We created 4 different advertisements to target these specific groups as a means of purposive sampling to obtain a more representative sample. Details on these advertisements and the specific targeting criteria can be found in Multimedia Appendix 1. We then created multiple versions of the same survey, each with its own unique link, to track the respondents recruited by each advertisement.

Daily advertisement spending was increased or decreased for each ad to increase or decrease the number and frequency of advertisements shown and, consequently, the rate at which individuals from specific demographics were being recruited. Advertisements ran for a total of 40 days, during April and May 2018, until the cutoff date for recruitment. Owing to the length and format of the survey, advertisements were targeted to desktop users but not mobile device users, as we believed the survey would be easiest to complete on a full-screen device.

For geographic targeting, we created an ad (rural ad) that targeted people aged 35 to 65 years and older in rural postal codes in NL. For the purpose of this study, we used Canada Post’s classification system to determine whether an area was rural or urban, with rural areas being indicated by having a 0 for the second character in the postal code [13]. Facebook allows advertisements to be targeted geographically by forward sortation area (the first 3 characters of the postal code), and so we targeted the rural ad to all forward sortation areas in NL that contained a 0.

To target by age, we created an ad (age ad) that was shown to individuals aged 35 to 44 years. To target by gender, we created an ad (gender ad) that was only shown to men.

To target by education (education ad), we applied exclusion criteria rather than inclusion criteria for ad targeting. We excluded individuals with higher levels of education so that the ad was only shown to people who had graduated high school, had completed some high school, or had not specified their level of education. We targeted by exclusion because educational attainment is optionally self-reported and so we believed it was less likely to be reported accurately by Facebook users. Applying inclusion criteria would have been more specific but it would have excluded an estimated 36,000 Facebook users in our study population who did not specify their level of education, leaving a target audience of only 18,000 Facebook users who had specified they were high school grads or had some high school (estimate obtained using Facebook ad manager).

#### Exclusion Criteria

Respondents (those who submitted the survey) were excluded from the sample if they did not meet all of the inclusion criteria or if they had more than 10 missing variables on the remainder of the survey (not including questions on screening history or any text-based responses, as these might not have been relevant for all respondents). The age and postal code of each respondent was checked to confirm that they met the inclusion criteria for the study. If they did not, or this information was not provided, the respondents were excluded.

### Statistical Analysis

Data analyses comprised the following 3 major aspects: (1) data cleaning and checking, (2) descriptive analysis of Facebook ad metrics and costs to determine cost per recruit, and (3) descriptive analyses to provide an overview of our sample and univariate analysis to examine whether the distributions of the sample’s sociodemographic and selected health characteristics were consistent with the target population. Census data were obtained from Statistics Canada for the 2016 census of the NL population for people aged 35 to 74 years [14]. The NL Centre for Health Information (NLCHI) provided data from the 2016 cycle of the Canadian Community Health Survey (CCHS) on NL residents aged 35 to 74 years. All statistical analyses were conducted using Microsoft Excel Version 16.17.

#### Campaign and Recruitment Measures

We investigated and reported advertisement campaign parameters, including the number of impressions, paid reach (unique individuals who saw the advertisement), unique link clicks, link clicks, ad spend, cost of administering the advertisements, cost per click, ad spend per recruit (only cost of advertisement included, which was used to compare results from different targeted advertisements), and cost per recruit (including ad spend and administrative costs). All costs are reported in Canadian dollars.

With respect to response rates, the number of unique site visitors could not be determined with Google Forms and so we used the numbers of unique link clicks provided by Facebook’s ad manager. Because the advertisement link directed people to the first page of the survey, the number of unique link clicks on the ad over the number of unique individuals who saw the ad (Facebook’s *paid reach* metric) will be considered the landing page view rate. Completion rate was defined as the number of recruits in our final sample over the unique link clicks. Google Forms does not track number of surveys started—only the number of surveys submitted—and there is no way to prevent, or identify when there are, multiple entries from the same individual, unless they were precise duplicates.

#### Feasibility

Feasibility was assessed both subjectively (the ease of conducting research and time commitment) and objectively (costs per recruit). Recruitment costs included both advertising costs and administrative costs during the entire recruitment period. We then compared this cost per recruit with an estimate provided by a local university-based research support unit for what they would have charged to conduct a postal survey using RDD sampling and telephone recruitment.

#### Representativeness

Representativeness of the Facebook sample was assessed by comparing sociodemographic characteristics of participants with the underlying population, obtained from the 2016 Census of the NL population between the ages of 35 and 74 years. These characteristics were as follows: age, gender, rural and urban geography, education, and household income. To further assess the representativeness of our sample, selected health characteristics were compared with the underlying population, using data from the 2016 CCHS of the NL population aged between 35 and 74 years. These characteristics were as follows: prevalence of arthritis, prevalence of diabetes, perceived health, having a regular health care provider, time since last seasonal flu shot, smoking status, body mass index (BMI), and whether they have ever had a colonoscopy or sigmoidoscopy.

Goodness-of-fit chi-square tests were conducted for each indicator to compare frequency counts from our sample with the expected relative frequency of the population to determine if the distribution of the Facebook sample was statistically consistent with the population. For the purpose of this analysis, *P*<.01 was considered to indicate rejection of the null hypothesis at significance level .01 (H_0_: distribution consistent with population; H_a_: distribution not consistent with the population). Likewise, *P*>.01 indicates failure to reject the null hypothesis at significance level .01, meaning that we can assume the sample distribution is consistent with the census distribution (ie, representative).

We then conducted post hoc tests for characteristics that had more than 2 categories using the methods described by Beasley and Schumacker [15] to find adjusted residuals (*Z*) and identify which categories were and were not consistent with the population. If the adjusted residual was −2.58≤Z≤2.58, the observed frequency for that category was considered similar to that expected under the null hypothesis at significance level .01 (ie, that the category was representative).

To determine the magnitude of difference between the sample distribution and the population, Cramer V posttest for effect size was calculated and interpreted as per Cohen [16], where values of 0.10, 0.30, and 0.50 corresponded to small, medium, and large effect sizes. We considered a characteristic to be *representative* of the population if (1) the chi-square test showed no statistically significant difference or, in case that there was a statistically significant difference, if (2) the Cramer V (df*=1) is less than 0.10. If Cramer V (df*=1), is between 0.10 and 0.20, we considered this small to medium effect size as *practically representative* of the population.

#### Targeting

The effectiveness of targeted advertisements at increasing representation of targeted demographics was assessed by comparing the distribution of the sample including the respondents from the targeted ad (observed) with the distribution of the sample excluding the respondents of the targeted ad (expected), using a chi-square statistical test at significance level of .01. If the targeted advertisement made a statistically significant change in the distribution that was closer to the target distribution of the underlying population, we considered the targeted advertising to be effective. We then compared advertisements based on dollars spent per recruit on advertising (ad spend per recruit).

### Ethical Considerations

Ethical approval was obtained from the NL Health Research Ethics Authority. Consent was obtained before individuals could begin the survey and if it was not provided, participants were redirected away from the survey. The survey itself was anonymous and all efforts were made to maintain confidentiality of individuals who participated. Facebook was used only to advertise and direct interested individuals to the survey link, which was hosted with Google Forms. Data were securely kept on password-protected computers and cloud storage accounts.

## Results

### Statistical Analysis

#### Campaign and Recruitment Measures

**Figure 2:**
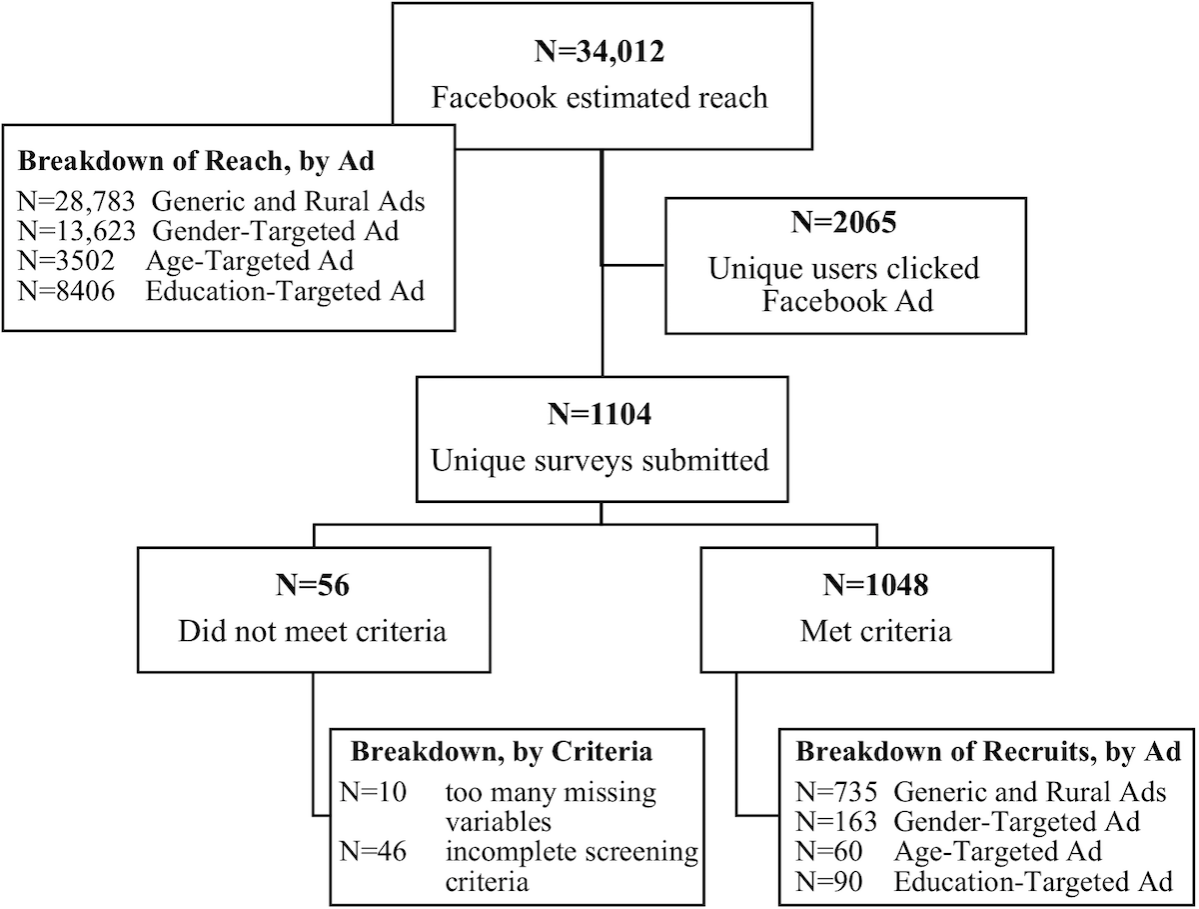
Details of the Facebook advertising and recruitment process. Note that the total estimated reach is lower than the sum of the number reached by individual advertisements, as some people were reached by multiple advertisements.

There are numerous metrics for measuring Facebook ad performance. The primary metrics and results are in Table 1. The cost of Facebook advertising was Can $1750 and the cost of a graduate student to administer this survey over the 40 days was approximately Can $539 (24.5 hours work). Figure 2 details the recruitment and selection process. The final sample had 1048 recruits, at an average cost per recruit of Can $2.18 (Can $1.67 ad spend per recruit). Administrative duties included creating the advertisements, responding to comments and questions, answering emails, and reviewing results and optimizing advertisements and targeting.

It was costlier to recruit men than women, with an average Can $3.63 ad spend per man recruited versus Can $1.69 ad spend per woman recruited. Considering all advertisements, women were more likely to be shown the ad than men (reach=21,416 for women vs 11,188 for men), were more likely to click on the link—the landing page view rate was 7.10% (1522/21,416) for women versus 4.16% (465/11,188) for men, and cost less per link click (Can $0.64 ad spend for women vs Can $1.16 ad spend for men). Women (799/21,416, 3.77%) who saw that ad were more likely than men (242/11,188, 2.16%) to complete it (number of recruits over paid reach). However, the actual completion rate—the number of recruits per unique clicks on the ad link—was similar for women and men (799/1522, 52.50% and 242/465, 52.0%, respectively).

To provide a brief understanding of the sampling frame and size of the NL population based on the 2016 census, there were 295,300 people aged between 35 and 74 years in NL [14]. The estimated reach provided to us by Facebook’s ad management tool was 110,000 active users aged 35 to 65 years and older. As Facebook’s ad manager tool does not provide the option to set the age range to between 35 and 74 years, we cannot provide an accurate estimate of the percentage of the population that is represented in our sampling frame.

**Table 1:** Facebook advertising campaign and recruitment metrics.

Metric		Women	Men	Unknown	Total
**Delivery^a^, n (%)**					
	Impressions^b^	85,693 (64.91)	41,370 (31.34)	4958 (3.76)	132,021 (100)
	Paid reach^c^ (No. of unique Facebook users)	21,416 (62.97)	11,188 (32.89)	1409 (4.14)	34,012^d^ (100)
**Engagement^a^, n (%)**					
	Unique link clicks^e^	1522 (73.63)	465 (22.50)	80 (3.87)	2067 (100)
	Overall link clicks	1697 (73.27)	527 (22.75)	92 (3.97)	2316 (100)
**Costs, Can $**					
	Ad spend	1084.63	609.60	55.77	1750
	Administrative costs of recruitment^f,g^	269.50	269.50	—^h^	539
**Performance**					
	Recruits, n (%)	799 (76.24)	242 (23.09)	7 (0.67)	1048 (100)
	Landing page view rate (unique link clicks/paid reach)^g^, %	7.10	4.16	5.68	6.08
	Completion rate (recruits/unique link clicks)^g^, %	52.5	52.0	—	50.7
	Ad spend per recruit^g^, Can $	1.36	2.52	—	1.67
	Cost per recruit^g^, Can $	1.69	3.63	—	2.18

^a^Metrics are estimated by Facebook.

^b^Impressions: the number of times any part of an ad appears on the user’s screen.

^c^Paid Reach: the number of unique people who have seen the advertisement.

^d^The total of paid reach is listed as 34,012 in the Facebook Ad Manager data tables, even though the sum of the 3 categories (women, men, and unknown) is 34,013. This has been left unaltered for transparency.

^e^Link Clicks: the number of clicks on the ad’s destination link to the survey.

^f^Administrative costs (Graduate student’s time: 24.5 hours or approximately 4.3 hours per week) include creating the advertisements, responding to comments/questions, answering emails, reviewing metrics and response to optimize advertisements.

^g^These metrics are not provided by Facebook but were calculated by the authors.

^h^Not applicable.

#### Feasibility

Economic considerations suggest Facebook advertising is a feasible means to recruit survey participants. The research was very easy to conduct, manageable by 1 graduate student as a research assistant committing 24.25 hours over 40 days. This method is also feasible for rapid recruitment of a large sample, with n=1048 participants being recruited in only 40 days and at a total cost of Can $2289 or Can $2.18 per recruit (excluding administrative costs, Can $1.67 ad spend per recruit). In comparison, we were quoted Can $21,316.05 as an estimate to contact 600 people with the anticipation of a final sample size of n=400 (Can $35.52 cost per recruit) using RDD. This considerably lower cost provides preliminary evidence to suggest that Facebook may be an economical recruitment method.

#### Representativeness

The sociodemographic characteristics of the sample are reported in Table 2. There was a statistically significant difference (*P*=.003) between the sample and population distributions for age. However, posttests showed a small effect size (V=0.145) between the 2 distributions. Post hoc analysis of adjusted residuals shows that the only age group that was not consistent with the population was the group aged 60 to 64 years, which was over-represented in our sample (*Z*=2.72: sample proportion is 180/1048, 17.17% vs population proportion is 14.23%). Considering the variation was small and was largely because of 1 age group, we concluded the age distribution of this sample is *practically representative*. Looking at the distribution of rural and urban recruits, there was no statistically significant difference between the sample and target population distributions at significance level .01 (*P*=.02), thus suggesting the distribution of rural and urban recruits is *representative* of the population distribution.

The distribution of annual household income was not consistent with the population (*P*<.001). Posttests showed a small to medium effect size (V=0.188). Post hoc analysis of adjusted residuals indicates that the variation is largely because of under-representation of individuals with household incomes under Can $30,000 (Z=−3.48: sample proportion is 141/942 [14.8%] vs population proportion is 19.84 *%*) and over-representation of individuals with household incomes between Can $50,000 and Can $59,999 (*Z*=3.43: sample proportion is 87/942, [10.2%] vs population proportion is 7.20%). All other income groups were consistent with the population. This, therefore, suggests that income is *practically representative* of the population.

**Table 2:** Comparison of selected sociodemographic characteristics of the Facebook sample with the underlying population of Newfoundland and Labrador from the 2016 census.

Characteristic		Facebook, n^a^ (%)	2016 census, %	Goodness-of-fit, χ^2^ (df)^b^	*P* value^b^	Cramer V (effect size)^c^	Adjusted residual, *Z*^d^	Interpretation^e^, representative?
**Gender**				267.1 (1)	<.001	0.507 (large)	—^f^	No
	Men	242 (23.25)	48.56	—	—	—	—	—
	Women	799 (76.75)	51.44	—	—	—	—	—
**Age (years)**				21.9 (7)	.003	*0.145 (small-medium)*	—	*Practically*
	35-39	96 (9.15)	10.41	—	—	—	*−1.32*	—
	40-44	98 (9.34)	11.68	—	—	—	*−2.35*	—
	45-49	125 (11.92)	13.09	—	—	—	*−1.12*	—
	50-54	159 (15.16)	14.43	—	—	—	*0.68*	—
	55-59	170 (16.21)	14.59	—	—	—	*1.49*	—
	60-64	180 (17.16)	14.23	—	—	—	2.72	—
	65-69	147 (14.01)	12.70	—	—	—	*1.29*	—
	70-74	73 (6.96)	8.86	—	—	—	*−2.16*	—
**Geography**				*5.4 (1)*	*.02*	*0.073 (small)*	—	*Yes*
	Rural	470 (44.85)	48.42	—	—	—	—	—
	Urban	578 (55.15)	51.58	—	—	—	—	—
**Education**				248.8 (1)	<.001	0.488 (large)	—	No
	No postsecondary	237 (22.72)	47.10	—	—	—	—	—
	Postsecondary	806 (77.28)	52.90	—	—	—	—	—
**Household income, Can $**				33.6 (5)	<.001	*0.188 (small-medium)*	—	*Practically*
	Less than 30,000	141 (14.8)	19.84	—	—	—	−3.48	—
	30,000-49,999	188 (19.8)	18.01	—	—	—	*1.26*	—
	50,000-59,999	87 (10.2)	7.20	—	—	—	3.43	—
	60,000-79,999	140 (14.7)	12.82	—	—	—	*1.62*	—
	80,000-99,999	114 (12.0)	10.36	—	—	—	*1.55*	—
	More than 100,000	272 (28.6)	31.76	—	—	—	*−1.75*	—

^a^Totals may not match because of missing responses.

^b^Italicized chi-square and *P* values indicate failure to reject null (H_0_: distribution consistent with population) at significance level .01, that is, consistent with census distribution.

^c^Italics indicate if Cramer V suggests the distribution is representative (Cramer V≤0.10) or practically representative (0.10<Cramer V≤0.20). Cramer V effect size indicates the size of the difference between the sample and population, with smaller being more representative.

^d^Italicized *Z* indicates post hoc adjusted residual Z is −2.58>Z<2.58, meaning observed number of cases is statistically similar to what would be expected if null hypothesis was true, at significance level of .01, that is, consistent with census proportion for category.

^e^Representativeness decision based on authors’ interpretations of χ^2^, post hoc adjusted residuals (*Z)*, Cramer V effect size, and practical significance.

^f^Data not applicable.

**Table 3:** Comparison of selected health characteristics of the Facebook sample and the 2016 Canadian Community Health Survey for Newfoundland and Labrador (Canadian Community Health Survey data provided by the Newfoundland and Labrador Centre for Health Information).

Characteristic		Facebook, n^a^ (%)	CCHS^b^ 2016, %	Goodness-of-fit, χ^2^ (df)^c^	*P* value^c^	Cramer V, (effect size)^d^	Adjusted residual, *Z*^e^	Interpretation^f^, representative?
**Has arthritis**				344.4 (1)	<.001	0.573 (large)	—^g^	No
	Yes	89 (8.48)	35.98	—	—	—	—	—
	No	960 (91.52)	64.00	—	—	—	—	—
**Has diabetes**				9.7 (1)	.002	*0.096 (small)*	—	Yes
	Yes	95 (9.06)	12.20	—	—	—	—	—
	No	954 (90.94)	87.80	—	—	—	—	—
**Self-perceived health**				154.7 (4)	<.001	0.384 (medium-large)	—	No
	Excellent	93 (8.87)	17.92	—	—	—	−6.92	—
	Very Good	421 (40.17)	42.24	—	—	—	*−1.03*	—
	Good	373 (35.59)	22.78	—	—	—	8.69	—
	Fair	144 (13.74)	11.76	—	—	—	*1.86*	—
	Poor	17 (1.62)	5.30	—	—	—	−5.17	—
**Last flu shot^h^**				207.0 (3)	<.001	0.449 (medium-large)	—	No
	<1 year ago	461 (44.89)	29.57	—	—	—	9.03	—
	1 year to <2 years ago	62 (6.04)	7.35	—	—	—	− *1.55*	—
	2 years ago or more	215 (20.93)	14.21	—	—	—	5.72	—
	Never	289 (28.14)	48.87	—	—	—	−9.50	—
**Has regular health care provider**				*0.7 (1)*	*.94*	*0.025 (small)*	—	Yes
	Yes	951 (90.83)	90.90	—	—	—	—	—
	No	96 (9.17)	9.10	—	—	—	—	—
**Smoking status**				20.3 (1)	<.001	*0.141 (small-medium)*	—	Practically
	Daily or Occasional	182 (17.86)	23.88	—	—	—	—	—
	Do not smoke	837 (82.14)	76.12	—	—	—	—	—
**Body mass index**				18.7 (2)	<.001	*0.135 (small-medium)*	—	Practically
	Normal to underweight	246 (23.93)	27.06	—	—	—	—	—
	Overweight	353 (34.34)	37.64	—	—	—	—	—
	Obese	429 (41.73)	35.30	—	—	—	—	—
**Has ever had a colonoscopy or sigmoidoscopy**				1.2 (1)	*.27*	*0.034 (small)*	—	Yes
	Yes	460 (43.89)	42.20	—	—	—	—	—
	No (or no answer)	588 (56.11)	57.80	—	—	—	—	—

^a^Totals may not match because of missing responses.

^b^CCHS: Canadian Community Health Survey.

^c^Italicized chi-square and *P* value indicate failure to reject null (H_0_: distribution consistent with population) at significance level .01, that is, consistent with census distribution.

^d^Italics is used to indicate if Cramer V suggests the distribution is representative (Cramer V≤0.10) or practically representative (0.10<Cramer V≤0.20). Cramer V effect size indicates the size of the difference between the sample and population, with smaller being more representative.

^e^Italicized *Z* indicates post hoc adjusted residual *Z* is −2.58>Z<2.58, meaning observed number of cases is statistically similar to what would be expected if null hypothesis was true, at significance level of .01, that is, consistent with census proportion for category.

^f^Representativeness decision based on authors’ interpretations of χ^2^, post hoc adjusted residuals (*Z*), Cramer V effect size, and practical significance.

^g^Data not applicable.

^h^Question asked slightly differently between CCHS and our Facebook Study, but results are unlikely to vary.

Gender was not representative of the population (*P*<.001; V=0.507), with women being considerably over-represented (see Table 2). Likewise, the distributions of high and low education in our sample were not consistent with population distributions (*P*<.001; V=0.488).

Table 3 shows the distribution of health characteristics of the sample in comparison with the underlying population. Our sample was representative of the proportion of people with diabetes in the population (*P*=.002; V=0.096), of the proportion of people with regular health care providers (*P*=.94), and of ever having a sigmoidoscopy or colonoscopy (*P*=.27; V=0.034). The sample was *practically representative* of smoking status (*P*<.001; V=0.14) and BMI (*P*<.001; V=0.135). The sample was not representative of the proportion of people with arthritis (*P*<.001; V=0.573), of perceived health status (*P*<.001; V=0.384), and of flu shot frequency (*P*<.001; V=0.449).

Caution should be used in considering the results for BMI mentioned in Table 3, as there is a potential data quality error for which we adjusted through imputation. When asked about body weight, participants were asked to *please specify pounds or kilograms*, as we presumed this choice would make it easier for the user to specify their weight. Unfortunately, a considerable number (n=225) provided a number but no units. To correct for this, any values that were equal or greater than 91 were assigned as pounds (n=221) and any values less than 91 were assigned as kilograms (n=4). This decision was made because pounds are most commonly used to specify weight in NL (and 95% of our sample that specified units did so in pounds). The cutoff of 91 was chosen because this was the sample’s minimum value for weight in pounds. We then determined if the distribution of BMI class for the participants whose BMI was calculated based on our correction (Group 1) was similar to the distribution of the group of participants who needed no corrections to calculate BMI (Group 2). Using an independent samples chi-square test, we found no statistically significant differences between Groups 1 and 2 (χ^2^_2_=1.1; *P*=.57). On the basis of this test, we deemed our method of imputing units onto values appropriate because it did not change the distribution of BMI, and for this reason we decided to retain the participants with corrected weights in the sample.

#### Targeting

The results of the targeted advertisements are shown in Table 4, presenting sociodemographic distributions of recruits, by the advertisement used to recruit them.

**Table 4:** Sociodemographic characteristics of samples recruited with each Facebook advertisement and the costs associated with each advertisement.

Characteristic		Advertisement source			
		Generic and rural^a^	Gender (men)	Age (years; 35-44)	Education (low)
**Gender, n^b^ (%)**					
	Men	86 (11.7)	150 (92.0)	2 (3)	4 (4)
	Women	644 (87.6)	13 (8.0)	57 (95)	85 (94)
	Other/not specified	5 (0.7)	—^c^	1 (2)	1 (1)
**Age (years), n^b^ (%)**					
	35-39	51 (6.9)	10 (6.1)	31 (52)	4 (4.4)
	40-44	61 (8.3)	5 (3.1)	27 (45)	5 (5.6)
	45-49	94 (12.8)	18 (11.0)	2 (3)	11 (12.2)
	50-54	127 (17.3)	20 (12.3)	—	12 (13.3)
	55-59	123 (16.7)	29 (17.8)	—	18 (20.0)
	60-64	131 (17.8)	38 (23.3)	—	11 (12.2)
	65-69	100 (13.6)	29 (17.8)	—	18 (20.0)
	70-74	48 (6.5)	14 (8.6)	—	11 (12.2)
**Geography, n^b^ (%)**					
	Rural	364 (49.5)	67 (41.1)	8 (13)	31 (34)
	Urban	371 (50.5)	96 (58.9)	52 (87)	59 (66)
**Education, n^b^ (%)**					
	No postsecondary	164 (22.5)	37 (22.7)	6 (10)	30 (33)
	Postsecondary	566 (77.5)	126 (77.3)	54 (90)	60 (67)
**Associated costs, Can $**					
	Ad spend	486.62 (rural ad); 426.58 (generic ad)	495.72	83.6	257.48
^ ^	Administrative cost^d^	378.02	83.83	30.86	46.29
	Total cost^e^	1291.22	579.55	114.46	303.77
^ ^	Ad spend per recruit	1.24	3.04	1.39	2.86
	Total cost per recruit	1.76	3.56	1.91	3.38

^a^The first sample source (ad—generic and rural) included recruits from both the generic ad and the rural ad. Owing to an error in the survey links used in the rural and generic advertisements, we were not able to distinguish which ad the participants had been recruited with, and so we had to present them together.

^b^Totals may not match because of missing responses.

^c^No participants in this category were recruited by this ad.

^d^Administrative costs for each ad was calculated by multiplying the total administrative cost for all advertisements (Can $539) by the fraction of total recruits recruited by that ad.

^e^Total cost was calculated by adding ad spend together with administrative costs.

##### Targeting by Geography

Respondents to our survey were representative of both rural and urban geographies in NL. Owing to an error in the survey link, we were not able to differentiate which respondents were recruited from the generic ad and which were recruited from the rural ad, as both advertisements directed people to the same survey instead of 2 different surveys as intended. The respondents to the generic and rural advertisements were evenly distributed between rural and urban regions (364/735, 49.5% and 371/735, 50.5%, respectively), whereas the other advertisements, combined, favored urban regions (207/313, 66%) over rural (106/313, 34%). This suggests, but does not confirm, that targeting by geography is effective, and the cost of these advertisements (Can $1.24 ad spend per recruit) was relatively low.

##### Targeting by Gender

The gender-targeted ad was effective for targeting men, although costlier. Without targeting, the sample would have been 10.5% men (92/878) and 89.5% women (786/878). With the gender-targeted ad, the final sample consisted of 23.25% men (242/1041) and 76.75% women (799/1041). Although the final sample did not achieve the same distribution as the census population, this showed that targeting by gender could increase the representation of the targeted gender in the sample in a statistically significant way (*P*<.001). However, the advertising costs were much greater than other targeted advertisements at Can $3.04 ad spend per recruit.

##### Targeting by Age

Without age targeting, the sample would have been 6.5% (65/988) of people aged 35 to 39 years and 7.2% (71/988) of people aged 40 to 44 years. With the age-targeted ad, the final sample was 9.16% (96/1048) of people aged 35 to 39 years and 9.35% (98/1048) of people aged 40 to 44 years. The difference was statistically significant (*P*=.005). The cost of age targeting (Can $1.39 ad spend per recruit) was not considerably more than the generic and rural targeted advertisements (Can $1.24 ad spend per recruit), and so this additional recruitment was effective and economical.

##### Targeting by Education

Without the education-targeted ad, 21.7% (207/952) of the sample had no postsecondary education and 78.4% (746/952) did. With the education-targeted ad, 22.72% (237/1043) had no postsecondary education and 77.28% (806/1043) did, but this difference was not statistically significant (*P*=.43). The cost to recruit these participants was considerably higher at Can $2.86 ad spend per recruit. Therefore, targeting by education was ineffective at increasing the proportion of participants who had lower levels of educational attainment.

## Discussion

### Principal Findings

This study is a novel investigation assessing whether or not Facebook advertising can be used to feasibly recruit a representative sample of middle-aged and older adults in the province of NL to complete a health survey. Representativeness was assessed by comparing numerous sociodemographic and health characteristics of our sample with the underlying population. Feasibility was assessed based on an assessment of costs, ease of use, and recruitment time. Moreover, this is the first Canadian study we know of to investigate Facebook advertising for the recruitment of adults aged up to 74 years; the vast majority of the national and international literature on social media recruitment focuses on the youth and young adults, with some focusing on middle-aged adult populations [5,10]. These results should be of considerable interest to academic researchers, community organizations, and governments, because they suggest this method can dramatically reduce barriers to conducting research with minimal sacrifices to representativeness.

### Feasibility

The research was very easy to conduct, manageable by 1 graduate student as a research assistant committing 4.3 hours per week. This method is also feasible for rapid recruitment of a large sample, with 1048 participants being recruited in only 40 days. A major advantage over other nonprobability-based sampling methods is that it was easy to use targeted advertisements to improve representativeness of a sample and to recruit hard-to-reach populations. There was also essentially no footwork involved, such as putting up recruitment posters, and we believe there was less selection bias than using email listservs or snowball sampling.

We also found that Facebook advertising is an economical means of recruiting participants for survey research. As anticipated, compared with the quoted cost of RDD recruitment, Facebook advertising was considerably less expensive. Granted, this is comparing our nonprobability-based sample with a probability-based sample. Though perhaps not an entirely fair comparison to make, it nonetheless demonstrates the considerable degree of reduction in cost. In comparison with other Facebook-recruited samples in the literature, our research appears more economical. A total of 2 systematic reviews exploring Facebook recruitment for health, medical, or psychosocial research found median recruitment costs of US $17.48 and US $14.41 per completing participant across various study designs, and the cost per recruit in cross-sectional surveys similar to our own was US $11.46 [5,10]. Although costs are not directly comparable because of differences in geography, topic of research, and targeted demographics, it appears that our Facebook recruitment was considerably more economical than in these previous studies. This could be because of the higher interest of the population in cancer as an issue that affects everyone. Many other studies focused on less prevalent health issues—such as vaccine-hesitancy, Human Immunodeficiency Virus–related knowledge, vertigo, endometriosis, or risky sexual behavior [5]—that might not elicit the same response as our research on cancer awareness and prevention did. This would increase the costs associated with advertising to reach a larger number of people to obtain the same sample size.

### Representativeness and Targeting

The Facebook-recruited sample was *representative* of the population with respect to age, geography, diabetes prevalence, having ever had a colonoscopy or sigmoidoscopy, and prevalence of having a regular health care provider, whereas it was *practically representative* with respect to income, smoking status, and BMI (see Tables 2 and 3). In their review, Thornton et al [5] found 16 Facebook-recruitment studies included a formal test of representativeness, with 9 testing the sample against the population of interest and 5 testing against traditional recruitment methods, such as phone and postal surveys, and 2 studies testing against both the population and traditional methods. Only 36% reported their samples were representative of their populations, but 86% were representative of samples recruited using traditional recruitment methods [5]. With 8 of 13 sociodemographic and health status variables representative or practically representative of the target population, our research further supports the conclusion that Facebook can yield a sample that is partially representative of the population. Targeted advertising was useful, but if we had begun targeted advertising earlier in the recruitment period and allocated more advertising dollars toward it, we believe we could have achieved a greater degree of representation from these groups.

#### Sociodemographic Characteristics

Targeting by age and gender was effective in increasing representation. Targeting men, although effective, was costlier. We suspect that targeting by geography helped increase the representation of rural participants in our study. However, because of the aforementioned error in the survey link for the rural ad, this is only a speculation. There was over-representation of people with higher levels of education, but targeting advertisements proved both costly and ineffective at improving representation of participants with lower levels of educational `attainment. We hypothesize this might be because targeting for education relies on the data that Facebook users optionally provide about their educational background. As such, it is possible that what is reported on a user’s Facebook profile won’t match their actual educational background. In contrast, age and gender targeting may have been relatively more effective because Facebook requires new users to report age and gender data when creating their Facebook account.

Lastly, even after specifically targeting men, our final sample had markedly more women than men. This is unsurprising because women are more likely to participate in health research [17]. The over-representation of women in this sample could be further explained by higher rates of Facebook use among women (60% women vs 40% men) in our targeted audience (information obtained on March 1, 2019 from Facebook’s audience insights tool) and because 63% of the people who saw the ad were women. Gender-representation in our sample might have been improved by increased spending on the ad targeted toward men, but it was 2.14 times costlier to recruit men than women (Can $3.63 vs Can $1.69 ad spend per recruit).

Overall, our difficulties recruiting certain demographics and obtaining a fully representative sample are common in other sampling methods [17-20]. Being under-representative of people in the lowest income bracket is typical across both random and nonrandom sampling methods [17,18,20]. This may be because of socioeconomic barriers, such as not being able to afford internet or landline phones, which could reduce their chances of selection. Furthermore, people with lower socioeconomic status may be less interested in participating in research [17,21].

#### Health Characteristics

The self-reported health of participants in our sample was not representative of the population, with more participants self-reporting their perceived health status as *good*, and considerably less self-reporting it as *excellent* or *poor*. However, our recruited sample appeared to be more health conscious than the general population, according to various health characteristics compared in our study. For instance, compared with the population, a higher proportion of our sample had a flu shot within the last year. Influenza vaccination uptake is known to be associated with positive beliefs toward prevention, trust in the health care system, socioeconomic status, and a number of other personal, intermediate, and structural factors [22]. Overall, we believe the general health of the sample was at least partially representative of the population, given that 5 out of the 8 assessed health characteristics were representative of the population (see Table 3).

### Strengths

Our study contributes to the evidence based on the use of social media for health research. We compared more sample characteristics with the population and used more formal tests of representativeness than many other studies have [1,5,10]. This method of Web-based recruitment works effectively in the context of NL. Furthermore, we have shown that Facebook can be used to recruit older adults to research surveys, where the vast majority of previous research on this subject has only considered younger populations. In addition, we went beyond simple tests of statistical significance by using Cramer V and post hoc adjusted residuals to add a greater depth to our analysis of how the sample distributions compare with the target population.

### Limitations

It should be noted that large sample size constrains the value of statistical tests because even small differences can appear *significant* when dealing with large sample sizes [23]. The feasibility assessment we employed was not rigorous, based mostly on subjective interpretations and on a cost comparison between our Web-based survey recruited through nonprobability-based sampling and a postal survey recruited through random sampling. As our analysis compares representativeness with the population (census or CCHS), it is not possible to directly compare representativeness of samples recruited with Facebook with different methods of recruitment, such as RDD. We encourage future research to consider conducting studies that allow for direct comparisons with probabilistic and nonprobabilistic sampling methods.

With respect to targeting advertisements by level of educational attainment, our use of exclusion criteria for targeting (excluding any Facebook users who have any higher level of education reported), rather than inclusion criteria (targeting only Facebook users who self-report education as high school or less), might have been too inclusive. The choice to use exclusion criteria was because there were approximately 36,000 people (Facebook-estimated potential reach) whose education was *unspecified* that would not have been shown the ad if we targeted for inclusion of *high school grad* or *some high school*. Although using inclusion criteria would have resulted in a smaller potential reach, it would have likely resulted in a higher proportion of recruits with lower levels of educational attainment. Future research employing Facebook advertising for recruitment might consider more specific targeting by using inclusion criteria for educational attainment.

There were 2 potential limitations with our data itself. First, the aforementioned issue with the rural-targeted ad. This limits our ability to conclude whether geographic targeting was effective, but we strongly believe that it is worth using geographic targeting for advertisements, which will be of benefit to researchers who want a geographically representative sample. The second data limitation is related to BMI calculations, already discussed above. The correction we employed (by imputing units for weight where they were not specified) did not affect the distribution in a statistically significant way, and so we believe this limitation has minimal impact on our findings.

### Considerations for Future Research

As our study examines effectiveness in a province with high Facebook usage, it may be more challenging to recruit representative samples in areas where Facebook use is lower. However, as NL has higher traditional landline usage than any other province (at 70% as of 2013), it is likely that traditional recruitment methods in other provinces would also yield less representative results [3]. For example, traditional landline usage rates are 43% in Quebec, 57% in British Columbia, and 61% in Ontario [3].

We believe there are many potential applications of Facebook advertising for researchers, governments, and community-based organizations who wish to learn about the populations they serve. Facebook advertising could also be used in qualitative research, sociological, and psychological research, where nonprobabilistic sampling is more common. Beyond research recruitment, we see Facebook advertising as having potential value in program planning and evaluation. For example, organizations planning a public health campaign may take a participatory approach by using Facebook advertising to reach individuals from their target population who they can ask to provide feedback on the campaign to optimize messaging and imagery.

Although the unique nature of Facebook advertising could warrant its own distinct sampling method classification, it is otherwise best described as nonprobabilistic purposive sampling. Although this technique is more common in qualitative research, it can be used to serve many purposes for both quantitative and qualitative studies [24]. A limitation of Facebook is that it is not possible to assess nonresponse bias, as there is little information on the nonrespondents, other than the age and gender of who has seen the advertisement. Another limitation of Facebook’s advertising tool is that it does not allow for specification of age targets above 65 years, thus limiting us to choosing the range of *35 to 65+* *years*.

Facebook’s health care research team recently published a scholarly paper suggesting the potential in using Facebook to advance the social determinants of health [25]. If Facebook has an interest in promoting health and health care research, we have several relatively simple recommendations on how this can be done. We strongly encourage Facebook to provide an option for random sampling of target audiences, as this will have considerable benefit in all areas of research—academic and otherwise. We further encourage Facebook to remove the *65+* *years* limitation for age targeting and allow users to specify any age range. This will be especially important as the number of older adults using Facebook continues to grow.

The greatest limitation of Facebook for research recruitment is, ironically, what makes it so useful as a marketing tool: Facebook’s targeting algorithm, which learns from people’s interactions with the ad, and then preferentially shows the ad to people with similar profiles. This produces an inherent sampling bias toward people who are more likely to respond to the advertisement. Therefore, because of the nonprobabilistic sampling nature of Facebook advertising, we caution against making certain inferences about the population—even if the sample is apparently representative of population demographic characteristics. Furthermore, given how the internet is constantly changing as technology grows, it is possible that this recruitment method may become more or less representative over time. Future changes to Facebook’s targeting algorithm may also impact its reliability in recruiting samples.

Researchers should carefully weigh the importance of having a probabilistic sampling method in achieving their research objectives with the importance of achieving a robust sample size within resource and time constraints. If researchers believe the latter is of higher importance, then Facebook advertising should be considered. It could be especially effective in comparison with other nonprobabilistic sampling methods; other research has noted that Facebook may be able to obtain more representative samples than other types of nonprobabilistic methods [19]. Moreover, probabilistic sampling does not inherently result in representative inferences, so although some may criticize nonprobabilistic sampling, it can yield valid inferences with proper weighting and adjustment [26].

Finally, the literature addresses many concerns and provides guidance and tools for researchers to deal with issues, such as data ownership, privacy, bias, and communication between participants and potential participants [19,27-29]. The pertinent issue that arose in our study was that of communication with and between participants. Anyone who sees the advertisement may comment on it and so researchers should carefully consider how they will interact with participants, particularly in response to questions and comments. For example, after noticing a few inappropriate comments on our own advertisements, we deemed it necessary to create a comments policy to guide how we handled responses including profanity, spam, irrelevant remarks, unauthorized medical advice, stigmatizing messages, and false statements. We published this policy as a *note* on our Facebook page and referred to it as necessary. In summary, there are numerous ethical concerns to social media recruitment and internet research which should be considered by future investigators using social media for recruitment.

### Conclusions

Overall, we achieved a partially representative sample, that is, it was representative or practically representative of 8 out of 13 sociodemographic and health characteristics assessed. Considering that this is a nonprobabilistic sampling method and the sociodemographic groups under-represented in our sample are commonly under-represented in probabilistic sampling methods, we believe these results are promising. These findings also suggest that purposively targeting subpopulations can improve representativeness. These findings suggest that Facebook advertising is a highly feasible and economical means of recruiting middle-aged and older adults for survey research. We, therefore, believe that Facebook advertising is useful for recruiting practically representative samples for many types of research and strongly encourage it in place of traditional nonprobabilistic methods where applicable. That said, there are inherent limitations to Facebook’s current targeting algorithm that limit its usefulness when probabilistic sampling is required for inferences. We urge researchers who wish to employ Facebook advertising for recruitment to be detailed and highly transparent when describing their methods.

## Appendix

Multimedia Appendix 1 Advertisement Campaign Details.

## Abbreviations

BMI: body mass index
CCHS: Canadian Community Health Survey
NL: Newfoundland and Labrador
NLCHI: Newfoundland and Labrador Centre for Health Information
RDD: random digit dialing
