# Supplementary material for: Using Facebook Advertising to Recruit Representative Samples: Feasibility Assessment of a Cross-Sectional Survey
Source: J Med Internet Res. 2019 Aug 19;21(8):e14021. doi: 10.2196/14021 (PMC6718121; doi:10.2196/14021)
Supplement: Multimedia Appendix 1 [file jmir_v21i8e14021_app1.pdf]

Multimedia Appendix 1. Advertisement Campaign Details

This is a Multimedia Appendix to a full manuscript published in the J Med Internet Res. For full copyright and citation information see http://dx.doi.org/10.2196/jmir.14021

Table 1. Detailed Advertisement Targeting Criteria

Targeting Criteria	Generic Ad	Gender Ad	Age Ad	Education Ad	Rural Ad
**Location**	Living in: Newfoundland and Labrador	Living in: Newfoundland and Labrador	Living in: Newfoundland and Labrador	Living in: Newfoundland and Labrador	Living in: A0A, A0B, A0C, A0E, A0G, A0H, A0J, A0K, A0L, A0M, A0N, A0P, A0R [a]
**Age**	35-65+	35-65+	35-44	35-65+	35-65+
**Gender**	All	Men	All	All	All
**Languages**	English (US), English (UK)	English (US), English (UK)	English (US), English (UK)	English (US), English (UK)	English (US), English (UK)
**Detailed Targeting[b]**	None	None	None	None	EXCLUDE people who match at least one of the following[c,d]:<br>• Associate degree<br>• College grad<br>• Doctorate degree<br>• In college<br>• In grad school<br>• Master's degree<br>• Professional degree<br>• Some college<br>• Some grad school

a. These are the rural postal code forward sortation areas in Newfoundland & Labrador.
b. Detailed Targeting is based on Demographics, Interests, Behaviours.
c. Targeting by Demographics > Education > Education Level.
d. Education Levels not excluded: High school grad, In high school, Some high school, and Unspecified.

Table 2. Images Used in Advertisements

Advertisement	Images Used	Notes
Generic	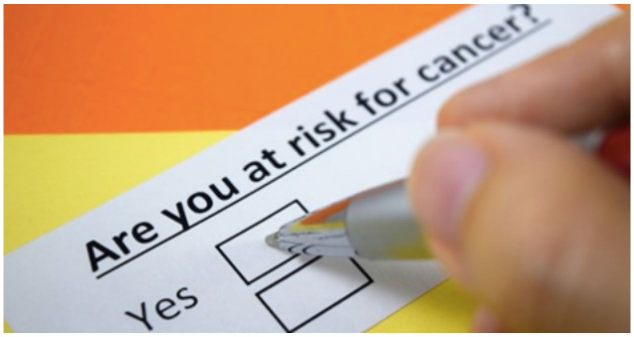	Cost Per Link Click: $0.44<br><br>Link Clicks: 1,097
Rural Ad (1)	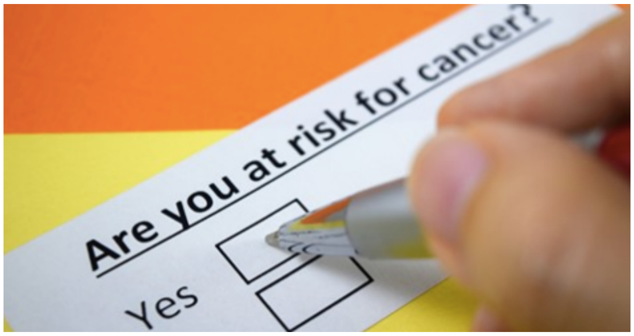	Cost Per Link Click: $0.87<br><br>Link Clicks: 311
Rural Ad (2)	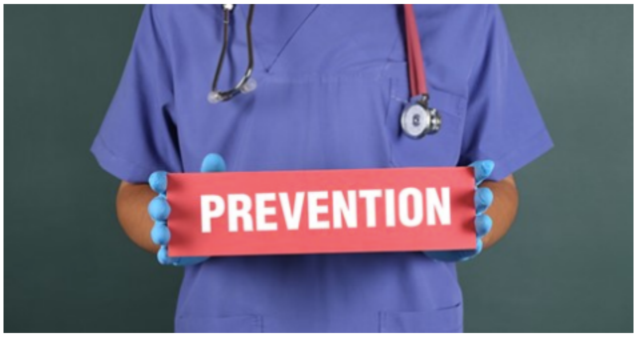	Cost Per Link Click: $0.97<br><br>Link Clicks: 159
Rural Ad (3)	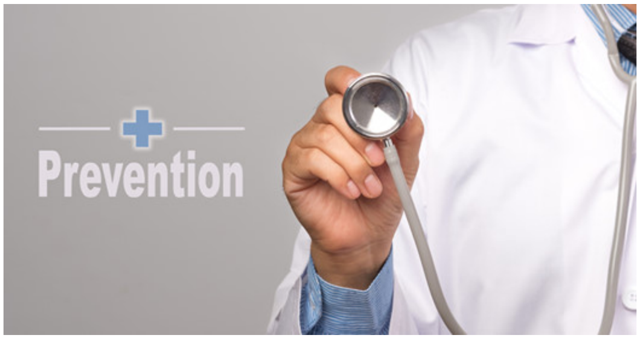	Cost Per Link Click: $2.41<br><br>Link Clicks: 2<br><br>This advertisment was discontinued after reaching 342 people (total ad spend was $4.82) due to the considerably higher cost.

Advertisement	Images Used	Notes
Gender Ad (1)	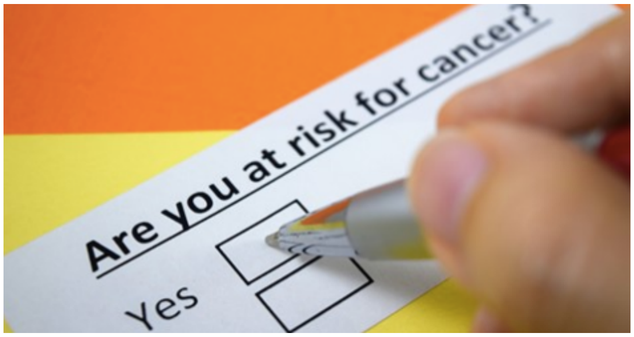	Cost Per Link Click: $1.30<br><br>Link Clicks: 252
Gender Ad (2)	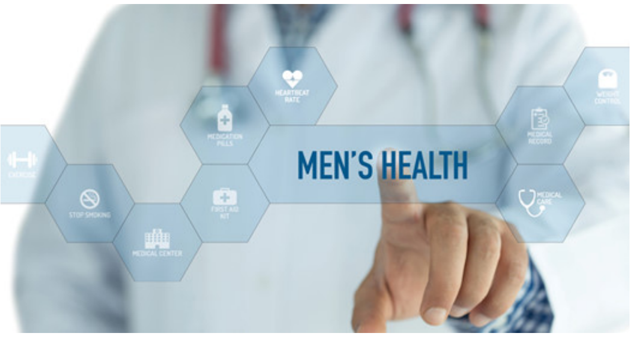	Cost Per Link Click: $1.90<br><br>Link Clicks: 88<br><br>This image was used in effort to reach more men, but the higher cost per link click suggests men were less responsive to this ad than Gender Ad (1).
Age Ad	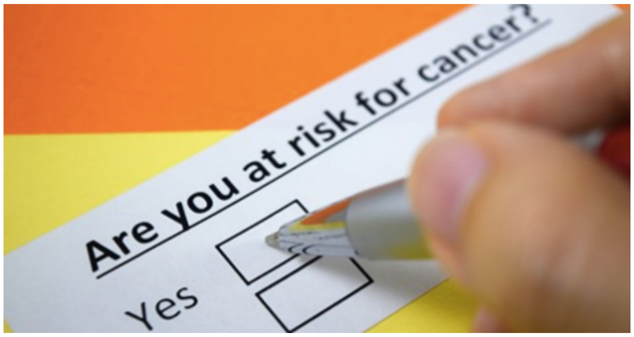	Cost Per Link Click: $0.73<br><br>Link Clicks: 114
Education Ad	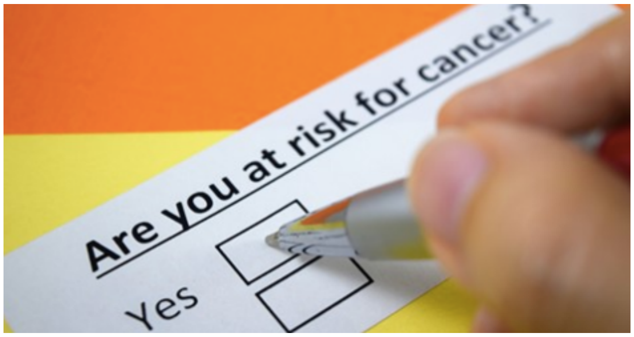	Cost Per Link Click: $0.88<br><br>Link Clicks: 293

Note. Various images were trialled in the Rural Ad and Gender Ad earlier in the recruitment period, but due to the better performance of the “are you at risk for cancer?” image, this was the imaged employed in ads later in the recruitment period. We recommend future researchers consider trialling various images in order to optimize their recruitment strategy.
